# Genomics and Functional Genomics of Malignant Pleural Mesothelioma

**DOI:** 10.3390/ijms21176342

**Published:** 2020-09-01

**Authors:** Ece Cakiroglu, Serif Senturk

**Affiliations:** 1 Izmir Biomedicine and Genome Center, Izmir 35340, Turkey; ece.cakiroglu@msfr.ibg.edu.tr; 2 Department of Genome Sciences and Molecular Biotechnology, Izmir International Biomedicine and Genome Institute, Dokuz Eylul University, Izmir 35340, Turkey

**Keywords:** malignant pleural mesothelioma, genomics, functional genomics, next-generation sequencing, RNAi and CRISPR screens

## Abstract

Malignant pleural mesothelioma (MPM) is a rare, aggressive cancer of the mesothelial cells lining the pleural surface of the chest wall and lung. The etiology of MPM is strongly associated with prior exposure to asbestos fibers, and the median survival rate of the diagnosed patients is approximately one year. Despite the latest advancements in surgical techniques and systemic therapies, currently available treatment modalities of MPM fail to provide long-term survival. The increasing incidence of MPM highlights the need for finding effective treatments. Targeted therapies offer personalized treatments in many cancers. However, targeted therapy in MPM is not recommended by clinical guidelines mainly because of poor target definition. A better understanding of the molecular and cellular mechanisms and the predictors of poor clinical outcomes of MPM is required to identify novel targets and develop precise and effective treatments. Recent advances in the genomics and functional genomics fields have provided groundbreaking insights into the genomic and molecular profiles of MPM and enabled the functional characterization of the genetic alterations. This review provides a comprehensive overview of the relevant literature and highlights the potential of state-of-the-art genomics and functional genomics research to facilitate the development of novel diagnostics and therapeutic modalities in MPM.

## 1. Introduction

Malignant pleural mesothelioma (MPM) is the cancer of the mesothelial cells of the pleura, the protective lining of the chest cavity. Although classified as a relatively rare cancer, MPM is the most common form of all mesotheliomas and is a highly aggressive and lethal cancer with nearly 25,000 deaths worldwide in 2018 [1,2]. Besides, the most recent annual statistics indicate that ~30,000 new cases [2] and ~3000 new patients are diagnosed globally and in the United States, respectively [3]. The primary etiological factor of MPM is the past occupational and environmental exposure to the exogenous factor asbestos, which accounts for ~80% of the diagnosed cases [4,5]. Notably, asbestos-related diseases cause nearly 93,000 deaths per year worldwide, reflecting the general health risks posed by asbestos fibers [4]. Although the use of asbestos has been regulated and restricted in Western countries, the global incidence of MPM is expected to increase over the next few decades due to the long latency period of the disease, estimated to be nearly 40 years after fiber exposure. Furthermore, the increasing use, import, and export of asbestos in many non-Western developing countries render MPM a continuing global health problem [4,5,6]. Exposure to the fibrous mineral erionite, radiation, and simian virus 40 are other recognized causes of MPM [5]. Additionally, rare familial forms of MPM possibly due to a genetic predisposition have been identified in several countries worldwide and particularly studied in the United States and Turkey, such as in the famous Cappadocia region [7,8,9,10].

There are three major histological subtypes of MPM: the epithelioid (50–70%), sarcomatoid (10–20%), and biphasic (20–35%). In epithelioid MPMs, tumor cells resemble the original mesothelial cells and typically present with pleural effusions and have the most favorable survival rates. Sarcomatoid MPMs, on the other hand, exhibit spindle cell morphology similar to sarcomas, are usually present with a pleural mass, and have the worst outcomes. Finally, biphasic or mixed MPMs have both epithelioid and sarcomatoid features [11,12]. The prognosis of MPM is generally poor, with a reported median overall survival of approximately one year after diagnosis, and there is no definitive cure for the disease [12]. Despite the recent developments in medical oncology, MPM is refractory to currently available multimodal therapeutic options such as chemotherapy, radiation, and surgical resection [13,14]. Besides, targeted therapy is not yet available for this deadly disease, even though several molecular pathways implicated in MPM have been identified to date. Of note, these pathways include cell cycle regulation, apoptosis, growth factor pathways, and angiogenesis [15]. Unfortunately, the clinical trials targeting these molecular processes have demonstrated limited, if not completely ineffective, efficacy against MPM [13,15]. Further studies are therefore needed for a thorough understanding of the biological basis of MPM, especially in the context of genetic, epigenetic, and transcriptomic alterations.

Interrogation of the functional significance of aberrations in cellular processes and signaling pathways is equally critical for the identification of novel molecular drivers, key vulnerabilities, and synthetic lethal interactions. Arguably, these comprehensive molecular studies may, in turn, lead to the development of effective targeted therapy strategies. In recent years, research on genomics and functional genomics of MPM, powered by next-generation molecular technologies such as DNA and RNA sequencing, high throughput RNAi (RNA interference), and CRISPR screens, and new preclinical models have begun to provide a deeper overview of the complex landscape of molecular mechanisms that contribute to MPM pathophysiology. The purpose of this review is to summarize the recent advances and significant contributions made by the researchers in the field of MPM research, in particular the next-generation and high throughput genomics and functional genomics studies. Further, we aim to address the fundamental molecular and cellular alterations underlying the pathogenesis and progression of MPM with particular emphasis on their possible implications for diagnostics and therapeutics.

## 2. Genomics of Malignant Pleural Mesothelioma

Malignant pleural mesothelioma is a heterogeneous cancer with complex underlying molecular alterations. To date, genomics analyses have revealed genetic, epigenetic, and transcriptomic deregulations in MPM.

### 2.1. Genomic Landscape of Recurrently Mutated Genes in Malignant Pleural Mesothelioma

The constant acquisition of genetic aberrations that facilitate oncogenic transformation, cellular proliferation, and invasion is a central mechanism in the course of tumorigenesis. Over the past two decades, efforts on comprehensive genomic landscape studies have contributed substantial insights into MPM biology. Owing to the rare nature and thus limited sample size, the chromosomal alterations identified and reported in the early genetics and the genomics studies of MPM were, although quite informative, usually repeated representations of recurrent mutations. Many of these genetic lesions are loss of function aberrations spurred by point mutations and/or copy number alterations (CNA) affecting multiple regions of the genome [16,17,18,19]. Unlike other cancers that are generally rich in oncogenic gain of function alterations, genomic losses in MPM carcinogenesis are more frequent. According to older karyotype and comparative genomic hybridization (CGH) studies conducted in the 1990s, losses in chromosome regions 1p, 3p, 6q, 9p, 13q, 15q, and 22q were common in MPM [20,21]. Subsequent studies have further confirmed that, along with losses, the most frequent genetic alterations in these chromosome regions are inactivating mutations found in the *NF2*, *CDKN2A*, *BAP1*, and *TP53* tumor suppressor genes, which play pivotal roles in restricting malignant transformation [16,17,18,19]. More recently, the advent of next-generation sequencing (NGS) technologies and the bioinformatics revolution fostered by large-scale genomics profiling initiatives, such as the global collaborative project The Cancer Genome Atlas (TCGA) [22], have enabled the discovery of novel genetic signatures and drastically enhanced our understanding and knowledge of MPM. As a consequence, novel mechanisms such as chromosome rearrangements, gene fusions, and splicing factor alterations have been identified as inactivation mechanisms for recurrently mutated genes [23,24,25].

#### 2.1.1. Frequent Loss of Function Alterations

The *BAP1* (BRCA1-associated protein 1) gene has been reported as one of the most frequently mutated genes in MPM, affecting more than half of the tumors [26]. In general, the functional loss of the *BAP1* gene, which is localized to the chromosomal region 3p31, a region that is frequently deleted in other cancer types as well, is caused by nonsynonymous somatic mutations, splice alterations, gene fusions, and copy number alterations [18,27,28]. The *BAP1* gene encodes a deubiquitylating enzyme with a ubiquitin carboxy-terminal hydrolase (UCH) domain that removes ubiquitin tags from numerous nuclear and cytoplasmic proteins, in particular histones, chromatin-associated HCF-1 (host cell factor 1); ASXL1, 2, and 3 (human homologs of Drosophila Polycomb group protein ASX); and IP3R3 (Inositol 1,4,5-Triphosphate Receptor, Type 3) [29,30,31,32]. BAP1 governs crucial cellular processes by regulating the expression of several target genes that control cell cycle progression, cellular proliferation and differentiation, Ca^2+^-signaling mediated apoptosis, ferroptosis, and energy metabolism [33,34,35,36]. Furthermore, BAP1 interacts with a number of nuclear proteins, such as BRCA1 (Breast Cancer Type 1) and BARD1 (BRCA1-associated RING domain protein 1), and assembles an active multiprotein complex that promotes homologous recombination and double-strand break repair, thereby maintaining genomic stability and functioning as a tumor suppressor [37]. Recently, heterozygous germline mutations eventually resulting in biallelic inactivation of the *BAP1* gene have been implicated in a rare condition named “BAP1 cancer syndrome”, which is characterized by the increased susceptibility to multiple cancers, primarily familial MPM, and uveal melanomas, and less frequently cutaneous melanomas, breast carcinomas, cholangiocarcinomas, sarcomas, basal cell carcinomas, renal cell carcinomas, and various types of CNS tumors [38,39,40,41,42]. It is of particular interest to mention here that germline mutations in DNA damage sensing and repair genes, and other tumor suppressor genes may also increase the risk of MPM development. Indeed, ~10% of MPM patients are carriers of heritable mutations in these cancer susceptibility genes [43,44,45,46].

Homozygous deletions of the CDKN2A (cyclin-dependent kinase inhibitor 2A) locus, specifically 9p21, have been demonstrated to be frequent in many primary tumor types of different origin. Similarly, *CDKN2A* is one of the most frequently inactivated tumor suppressor genes in MPM, deleted in about half of the cases [17]. Although less frequent, the complete genetic loss of INK4 locus, which is typically characterized by the co-deletion of *CDKN2A* and its syntenic gene *CDKN2B* (cyclin-dependent kinase inhibitor 2B), is also observed in MPM [21]. The human *CDKN2A* gene encodes two distinct tumor suppressor proteins resulting from translation of the common second exon in alternative reading frames (ARFs), namely, alpha and beta. Specifically, the alpha transcript encodes p16INK4A, while the product of the beta transcript is p14ARF. Besides, the *CDKN2B* gene codes for the p15INK4B protein. p14ARF is involved in the regulation of cell cycle progression and apoptosis, mainly by antagonizing the functions of MDM2 (Mouse Double Minute 2), the E3 ubiquitin-protein ligase responsible for p53 degradation [47]). Moreover, p16INK4A and p15INK4B are highly homologous proteins that are structurally and functionally similar cyclin-dependent kinase inhibitors (CDKIs). By inhibiting CDK4/6 activity, they cause hypophosphorylation and functional activation of pRb (the retinoblastoma tumor suppressor protein), thereby preventing cell cycle progression and proliferation [48]. Notably, the co-deletion of *CDKN2A* and *CDKN2B* genes is associated with a shorter overall survival rate in MPM. Emerging evidence suggests that MPM patients with the loss of function of p16INK4A and p15INK4B could benefit from cell cycle targeted therapies, in particular CDK4/6 inhibitors [49,50,51].

The other most frequent alteration in MPM involves the inactivation of the *NF2* (neurofibromatosis type 2) gene, a genetic event that was first reported in the mid-1990s and occurs in nearly half of the tumors [52]. The inactivation of the *NF2* gene is associated with homozygous chromosomal loss and focal deletions of the 22q12 locus. High rates of gene fusions; splicing defects; and frameshift, missense, and nonsense mutations that result in low expression or absence of the 70 kDa tumor suppressor protein Merlin (moesin-ezrin-radixin-like protein), the protein encoded by the *NF2* gene, have also been reported [53,54]. Merlin shares a similar domain organization with, and therefore belongs to, the Ezrin, Radixin, and Moesin (ERM) protein family [55]. As a multifunctional protein, Merlin mediates tumor-suppressive mechanisms by inhibiting malignant activities of cancer cells. To that extent, several studies have revealed that Merlin inhibits cell proliferation by limiting the signal transduction activities of several molecular pathways such as EGFR (epidermal growth factor receptor), PI3K (phosphoinositide 3-kinase), mTORC1/2 (mTOR complex 1/2), Hippo (Salvador-Warts-Hippo), Rac, and Ras GTPase effector pathways [56]. Besides, Merlin plays a crucial role in coupling a variety of membrane- and cytoskeleton-associated cellular processes such as cell morphology, adhesion, motility, and migration [55]. Finally, several lines of experimental evidence indicate that the functional loss of *NF2* gene in MPM often contributes to increased cell spreading and invasion through recruitment and hyperactivation of FAK (focal adhesion kinase) and c-Src (SRC proto-oncogene, non-receptor tyrosine kinase) signaling pathways [57,58]. Taken together, probing the clinical utility of inhibiting the cellular pathways associated with the loss of Merlin activity in MPM is worth exploring.

#### 2.1.2. Less Common Loss of Function Genetic Alterations

In addition to the loss of *CDKN2A*, *NF2*, and *BAP1* genes, an overwhelmingly well-known tumor suppressor *TP53* (tumor protein p53) gene is also, although to a lesser extent, inactivated in MPM. Mechanisms of inactivation include recurrent copy losses and nonsynonymous coding sequence mutations [23,59,60,61]. Inactivation of the *TP53* gene was found to correlate with a less favorable overall survival rate when compared to the wild type *TP53*, which would underscore that the MPMs with *TP53* mutations have a more aggressive phenotype [59,62]. Importantly, Hmeljak et al. has recently identified a novel molecule subtype of MPM characterized by recurrent *TP53* mutations, co-mutations of the *SETDB1* gene that encodes a transcriptional repressor histone methyltransferase, and genomic near-haploidization, as well as disctinct clinical attributes such as female predominance, younger age at diagnosis, and aggressive behaviour [62]. Other less frequently but recurrent significantly mutated genes documented in MPM include *LATS2* (large tumor suppressor kinase 2) and *SETD2* (SET domain containing 2). The *LATS2* gene aberrations, usually due to large deletions and point mutations, often co-occur with NF2 inactivation, a molecular event that appears to be MPM specific [63]. The eventual coinactivation of *LATS2* and *NF2* genes leads to deregulation of the Hippo pathway through enhanced cotranscriptional activity of YAP1 (Yes1 associated transcriptional regulator, also known as YAP), the downstream nuclear effector protein of the Hippo signaling pathway [63,64]. More recently, the *SETD2* tumor suppressor gene, along with other members of the SETD family genes, has been reported to be frequently inactivated in MPM through recurrent mechanisms of gene fusions, splice alterations, and nonsynonymous mutations [23,65]. The *SETD2* gene encodes a histone lysine methyltransferase that trimethylates Lys-36 of histone H3, an epigenetic modification associated with active transcription [66]. However, the functional consequences of SETD2 inactivation in MPM are not known. On the other hand, studies in human renal cell carcinomas suggest that the functional loss of *SETD2* gene, together with other chromatin remodelers, could lead to epigenome aberrations and pathological splicing events, as discussed in further detail below. Intriguingly, a recent work by Park et al. has found that SETD2 methylates the microtubules during mitosis and cytokinesis and the loss of this function results in mitotic spindle defects, impaired DNA repair, and genomic instability, which may explain, at least in part, the high frequency of aneuploidy observed in MPM [67].

#### 2.1.3. Gain of Function Mutations

Recurrent somatic copy number gains are less common in MPM. To date, chromosomal gains have been reported for multiple regions of the MPM genome, including 1q, 5p, 7p, 8q, and 17q. Interestingly enough, *TERT* gene (telomerase reverse transcriptase) copy number gains and hotspot promoter mutations constitute the first recurrent oncogenic gain of function alterations in MPM, which are generally observed in ~10–15% of all cancer types. The *TERT* gene encodes the catalytic subunit of the telomerase complex and the reactivation of TERT expression is a hallmark of cancer. Consistent with other cancer types, the spectrum of promoter mutations found in MPM result in augmented mRNA expression and the MPM patients with TERT promoter, *TP53* and *NF2* mutations have shorter overall survival [68,69]. Of note, the promoter mutations and copy number gains are not mutually exclusive, and the chromosomal amplifications alone do not account for transcriptional activation of the *TERT* gene (chr location: 5p15). Further, while mutually exclusive with BAP1 inactivation, *TERT* promoter mutations often co-occur with CDKN2A inactivation, which warrants the rationale for appraising the clinical competence of telomerase-inhibiting therapies, alone or in combination with CDK4/6 inhibitors [69,70].

Using low-pass whole genome sequencing (LP-WGS) on a set of 21 MPM cases with matched normal samples, Hylebos et al. identified novel copy number gains in the oncogenes such as *CD79B* (CD79b molecule, chr location: 17q23) and *MDM4* (MDM4 regulator of p53, chr location 1q32) [65]. Supported by their oncogenic roles in other cancers, MPM-specific functional understanding of these genetic alterations can make them attractive targets for MPM therapy. In one of the most comprehensive studies of MPM genomics, Bueno et al. have recently discovered recurrent gains in *RPTOR* (regulatory associated protein of mTOR complex 1, chr. location: 17q25), a gene with known oncogenic functions in colorectal cancer [23]. Further studies are needed to confirm or refute a similar biological function in MPM biology. Similarly, deregulated amplification of *MYC* gene (MYC proto-oncogene, bHLH transcription factor, chr location: 8q24), a genetic event that can be recapitulated in asbestos-induced MPMs of wild type murine models, has been reported in a small fraction of MPM cases (<5%) [71,72].

### 2.2. Novel Malignant Pleural Mesothelioma-Associated Mutations

High-throughput NGS technologies have revolutionized genomics studies and enabled the extensive characterization of cancer-specific somatic mutations at unprecedented resolution. Further, the arrival of these technologies has promoted the development of the public cancer genomics resources, such as the Catalogue of Somatic Mutations in Cancer (COSMIC), International Cancer Genome Consortium (ICGC), and The Cancer Genome Atlas (TCGA) [73,74,75]. Of these, the TCGA resource that is a collaboration between the National Cancer Institute (NCI) and National Human Genome Research Institute (NHGRI), has achieved a milestone in cataloging the clinical and genomics attributes of more than 20,000 primary cancer tissues and matched normal samples, across 33 types of cancer. This unified repository offers both controlled and open access to a comprehensive resource of genetic, epigenetic, transcriptomic, and proteomic data, enabling the scientific community to address unresolved questions and make seminal discoveries in cancer.

To further expand the knowledge on MPM biology, various research groups have conducted in silico analyses on TCGA and other open access multi-institutional MPM datasets. These efforts have identified novel genetic aberrations and confirmed the earlier findings on recurrent alterations [35,36,37,38,39,40,41,42]. The data suggest that commonly detected novel variations are related to cellular processes such as DNA repair, histone methylation, RNA metabolism, and angiogenesis. Significant molecular alterations affecting PI3K-Akt, Wnt, Hippo, mTOR, PDGF (platelet-derived growth factor), and FGF (fibroblast growth factor) signaling pathways are also described [23,25,59]. Included among the mutated genes in these pathways are *APC*, *ARID1A*, *BMPR1A*, *CHEK2*, *DVL1*, *EPHB1*, *FBXW7*, *KDR*, *MAP2K1*, *PDGFRA*, *PTEN*, *RB1*, *SMARCA4*, *SMARCB1*, *HRAS*, *NRAS*, and *WRN* [23,24,59,70,76,77], whose genetic alterations have also been found in lung adenocarcinomas [78,79,80], colorectal [81,82,83,84], and breast cancers [85]. Moreover, oncogenic mutations in *ABL1*, *EP300*, *PIK3CA*, *PIK3C2B*, *PTPN11*, and *SETDB1* genes [23,24,25,59,62], and novel gains in regions encompassing *RHEB*, *KDM5A*, *AXIN2*, *RICTOR*, *TRIO*, and *DVL1* genes were identified [24,65]. Furthermore, *TP53*, *PIK3CA*, and *KDR* mutations were clinically associated with significantly reduced overall survival [59]. Looking ahead, we believe that further studies based on high-throughput dataset analyses on published work or directly on publicly available genomics datasets will continue to make seminal contributions in shedding light on MPM pathobiology. In accordance with the ease of self-assessment and reporting of the TCGA dataset, we performed genomic analysis on the cohort of 87 MPM patient samples at The cBio Cancer Genomics Portal (cBioPortal) [86,87] and summarized the findings in Table 1.

### 2.3. Deregulations in Signaling Pathways, Cellular Processes, and Gene Expression

High-throughput transcriptomic profiling of MPM has revealed frequent deregulations in genes and signaling circuits that regulate cellular processes such as cell division and proliferation, DNA replication and repair, response to mitogens and stress, survival, apoptosis, and migration [23,88].

Growth factor signaling pathways contribute to MPM tumorigenesis, and they are recurrently deregulated in MPM. Mechanisms of deregulation involve sustained activation of autocrine and paracrine signaling [21]. Among others, dysregulation of receptor tyrosine kinase (RTK) signaling results in abnormal activation of the PI3K/Akt/mTOR, and MAPK pathways [62,89,90]. These pathways regulate critical biological processes such as transcription, translation, cell proliferation, growth, and migration that are often deregulated in MPM. Independent studies have reported a positive association between phosphorylation levels of Akt, and mTOR in more than half of the MPMs. Besides, the activation of PI3K/Akt/mTOR has been associated with the loss of PTEN expression, the negative regulator of PI3K-Akt pathway [91,92,93]. Furthermore, MAPK cascade has displayed increased activity of the intracellular signaling molecules in older and recent reports [62,94,95]. However, the activation status of this pathway failed to differentiate between malignant and nonmalignant cells, highlighting a complex regulation in MPM [89,95]. Consequently, implementing therapeutic interventions appear to be challenging with the current state of MPM knowledge and additional studies will be required to define the role of the MAPK pathway in MPM pathophysiology.

Increased activity of EGFR signaling pathway has also been reported in MPM [96,97,98]. However, unlike lung cancer, the mutation and expression status of EGFR correlated with better overall survival in MPM patients, which may explain why the Phase II clinical trials of Erlotinib and Gefitinib, the first generation EGFR tyrosine kinase inhibitors (EGFR-TKIs), did not yield significant responses [99,100]. Overall, available data suggest that targeting the EGFR pathway with EGFR-TKIs at this time would not be considered therapeutically relevant in MPM.

Pathological angiogenesis is a key mechanism in cancer cell proliferation, invasion, and metastasis, and is thus a hallmark of cancer [101]. Likewise, deregulated angiogenesis is an integral component of the highly aggressive behavior, and the chemotherapy-resistant nature of MPM [102,103]. This has been obtrusive by way of augmented expression of HIF-1α (hypoxia-inducible factor 1α) and elevated blood neovascularization as measured via vessel density [104,105]. Furthermore, deregulations in master regulators of angiogenesis, which include increased expression of VEGF (vascular endothelial growth factor) family proteins and their specific receptors (VEGFRs), and the downstream targets of VEGF/VEGFR signaling axis, mainly the RTK signaling mediators PKC (protein kinase C) and Akt, have been implicated in MPM tumorigenesis [106]. Similarly, deregulation of FGF and PDGF pathways by virtue of autocrine and paracrine signaling, have been associated with MPM cell survival and angiogenesis [107]. Despite the negative results with earlier antiangiogenic therapies, newly designed clinical studies targeting VEGF and other growth factor signaling pathways, alone or in combination, are underway [105].

Frequent acquisition of genome-wide copy number alterations in MPM is linked to impaired microtubule and spindle dynamics and chromosome segregation, a key aspect of accurate cell cycle regulation and genome stability. The condensin complex, kinesin family proteins (KIFs), cyclins, and the MSAC (mitotic spindle assembly checkpoint) pathway regulate precise spindle organization and chromosome segregation during cell division [108,109,110,111]. While no mutations had been identified, important deregulations of these molecular networks were reported. Specifically, CNAP1, NCAPD3, and BRRN1 of the condensin complex, cyclin genes (*CCNA2*, *CCNB1*, *CCNB2*, and *CCNL2*), and kinesin family genes (*KIF14*, *KIF23*, *KIFC1*, and *KIF4A*) were significantly elevated in MPM [112]. Further studies have depicted increased expression of BUB1 (BUB1 mitotic checkpoint serine/threonine-protein kinase) and MAD2L1 (mitotic arrest deficient 2 like 1), the essential components of the MSAC pathway, which reportedly predicted poor prognosis in MPM [113]. Other MSAC components, such as cell cycle-regulated serine/threonine Aurora kinase family members AURKA and AURKB, and Survivin (also called baculoviral inhibitor of apoptosis repeat-containing 5 or BIRC5), which function in centrosome maturation and cytokinesis progression, were found upregulated and associated with poor outcomes [113]. These findings indicate that targeting the components of the microtubule network in MPM might be a viable option.

The epithelial–mesenchymal transition (EMT) is a vital cellular program wherein epithelial cells lose their polarized epithelial phenotype and acquire features inherent to mesenchymal-like cells, characterized by increased expression of extracellular matrix (ECM) proteins, enhanced motility, invasiveness, metastasis, and resistance to apoptosis [114]. The EMT process plays an essential role in MPM pathogenesis and is associated with high-grade malignancy, thus poor prognosis. In this regard, while the epithelioid subtype displays features of epithelial cells, the sarcomatoid and biphasic histological subtypes are usually recognized as mesenchymal and intermediate phenotypes, respectively [115]. Many genes associated with reprogramming of cell–cell and cell–extracellular matrix interactions orchestrated by means of the EMT process are differentially expressed in MPM and correlate with worse survival. Interestingly, a substantial number of these genes such as matrix metalloproteases (*MMP3*, *MMP7*, and *MMP14*), integrins, and a group of cytoskeletal proteins and cell adhesion molecules (*ITGA3*, *ITGB4*, *VCAN*, *VIM*, *PECAM1*, *CDH1*, *CDH5*, and *CD44*), structural molecules, and modifiers of ECM (*LOXL2*, *BNC1*, *GREM-1*, *FBN2*, *THBS2*, *RASGRP3*, *ANXA6*, *ADAMTS6*, and *ADAM19*) and transcription factors (*SNAI1*, *SNAI2*, *ZEB1*, *TWIST1*, and *HMGA2*) are either significantly enriched in the gene signature or recognized as signaling modulators of the TGF-β (transforming growth factor β) pathway [19,62,112,116,117,118,119,120,121,122]. Indeed, a large body of evidence that accumulated over the last few years, suggests that the TGF-β signaling pathway is dysregulated in MPM. Thereof, a less favorable prognostic association has been identified between the EMT score of MPM and the activated TGF-β gene signature [119,123]. Taken together, further studies may provide the basis for assessing TGF-β pathway-specific inhibitors for targeted therapy in MPM, at least in those with high EMT scores.

A comprehensive genomic analysis has recently studied the differential gene expression profiles of 216 human primary MPM samples and their matched normal counterparts, one of the largest cohorts in recent years. RNA sequencing results identified a total of 430 differentially expressed genes: 189 upregulated and 241 downregulated. Consistent with previous reports, the EMT gene signature was able to define distinct molecular subgroups of MPMs. The most significantly upregulated mesothelial marker genes in the epithelial group included cytoskeleton, transmembrane, cell junction and, extracellular matrix assembly genes, e.g., *ELMO3*, *MSLN*, *ITLN1*, *SYT4*, *UPK3B*, *CLDN15*, *LRRN4*, *RSPO1*, *VTN*, and *KLK11*. On the contrary, EMT-positive marker genes, e.g., *LOXL2*, *VIM*, *ADAMTS6*, *CDH13*, *CDH17*, *SNAI2*, and *HMGA2*, were among the most significantly upregulated genes in the sarcomatoid group. Even though the functional role of many of these genes in MPM pathophysiology is yet to be confirmed, their differential expression could serve as a diagnostic, and therapeutic tool [23]. Remarkably, the expression ratio between the Claudin family gene *CLDN15*, which is normally downregulated in cells undergoing EMT, and the *VIM* gene, a marker for the mesenchymal state, was a significant discriminating signature between subgroups [124]. Observations like these indicate that incorporating genetic mutation profiles with gene expression deregulations would enable the identification of actionable alterations and facilitate the development of targeted therapies.

### 2.4. Epigenetic Deregulations

Functional deregulations in epigenetic modifications such as DNA methylation, post-translational histone modifications, and chromatin remodeling play diverse and critical roles in carcinogenesis. Molecular studies in MPM have identified aberrant epigenetic events affecting the pathogenesis of the disease. An early study by Christensen et al. compared the causality between asbestos exposure and epigenetic aberrations of MPM by particularly focusing on the promoter CpG methylation patterns of select tumor suppressor genes. A significant correlation between the asbestos burden and the methylation status of *CDKN2A*, *CDKN2B*, *APC*, and *RASSF1* tumor suppressor genes has been identified [125]. Consistent with their preceding report, Christensen et al. later noted that the global methylome profiles could effectively differentiate the normal pleura tissue from the malignant state and that they were predictors of patient survival [126]. Later, Goto et al. cross-compared epigenetic profiles of MPM and lung adenocarcinoma samples where high throughput analysis identified an average number of 387 hypermethylated genes in MPM samples vs 544 genes in lung adenocarcinoma. Among these genes, *TMEM30B*, *KAZALD1*, and *MAPK13* were specifically methylated in MPM tissues and have been proposed as putative diagnostic markers. Besides, a lower frequency of DNA hypermethylation has significantly correlated with a better clinical outcome [127]. Of particular interest, the authors of this study postulated that the two malignancies had distinct epimutation mechanisms, mainly induced by asbestos vs. smoking. In addition, the epigenetic silencing of *SFRP2* and *SFRP5* tumor suppressor genes, which encode secreted frizzled related proteins, has been observed in MPM samples [128]. These findings are of particular significance for two reasons: First, the promoter methylation profile of these genes could potentially serve as a plasma-based epigenetic biomarker. Second, SFRPs are antagonistic modulators of the Wnt signaling, and their downregulation via hypermethylation could, in part, explain the aberrant activation of the Wnt pathway in MPMs.

Recent studies have identified that methylation-mediated global dynamic changes in the transcriptome are correlated with increased expression of DNA methyltransferase genes, namely *DNMT1*, *DNMT3A*, and *DNMT3B*. Besides, the increased expression of these genes has been associated with shorter survival of MPM patients [129]. Other dynamic mechanisms of epigenetic modifications involve the acetylation and methylation of histones, and chromatin remodeling. The study by Goto et al. defined that a number of genes silenced in MPM samples were enriched for the repressive histone H3 lysine methylation (H3K27me3) mark [127], which is a downstream target of PRC2 (the polycomb repressive complex 2). Experimental evidence further indicated that increased H3K27me3 marks were associated with concomitant overexpression of EZH2 (enhancer of zeste homolog 2) and SUZ12 (SUZ12 polycomb repressive complex 2 subunit) which, by forming the PRC2 complex, contribute to tri-methylation of histone 3 lysine 27. Additional work revealed that increased expression of EZH2 and SUZ12 correlated with poor survival, highlighting the potential of therapeutic targeting of these deregulations in MPM [129]. In addition, Lafave et al. demonstrated that the loss of BAP1 in mice resulted in increased H3K27me3 and elevated expression of EZH2. Furthermore, pharmacological inhibition of EZH2 in *BAP1*-mutant MPM cell lines abrogated their growth in vitro and in vivo, suggesting that EZH2 could represent a genotype-specific vulnerability in MPM [130]. In strong support of these findings, Tazemostat, a first-in-class small-molecule inhibitor of EZH2 received accelerated FDA approval in January 2020 for the treatment of locally advanced or metastatic epithelioid sarcoma [131]. More importantly, it is currently undergoing clinical development for use in other tumor types, including MPM.

### 2.5. Deregulations of Non-Coding RNAs

Recent advances in RNA sequencing technology have revealed that only 1–2% of the human genome codes for proteins and the vast majority of the transcribed genome is composed of non-coding RNAs (ncRNAs) [132,133]. Although not translated into proteins, ncRNAs have been shown to play multidimensional roles in a wide range of biological processes such as activation or repression of transcription, regulation of mRNA translation, post-translational modifications, and chromatin remodeling [134,135]. Compelling experimental evidence suggests that ncRNAs have a profound impact on cancer hallmarks. Accordingly, their expression is frequently deregulated in carcinogenesis [136,137]. Two subclasses—microRNAs (miRNAs) and long non-coding RNAs (lncRNAs)—have been demonstrated to play important roles in cancer cell biology with tumor suppressor or oncogenic functions. Supported by extensive experimental evidence, ncRNAs possess potential clinical relevance as diagnostic biomarkers and molecular targets for many cancers including thoracic malignancies such as lung cancer and MPM [138,139,140]. Earlier MPM research on miRNAs and lncRNAs have been extensively reviewed elsewhere by Reid et al. and Singh et al., respectively [141,142]. Importantly, many significant studies on ncRNAs have been published after these reviews. Table 2 lists the most recent MPM studies on ncRNAs. 

## 3. Functional Genomics of Malignant Pleural Mesothelioma

**Figure 1:**
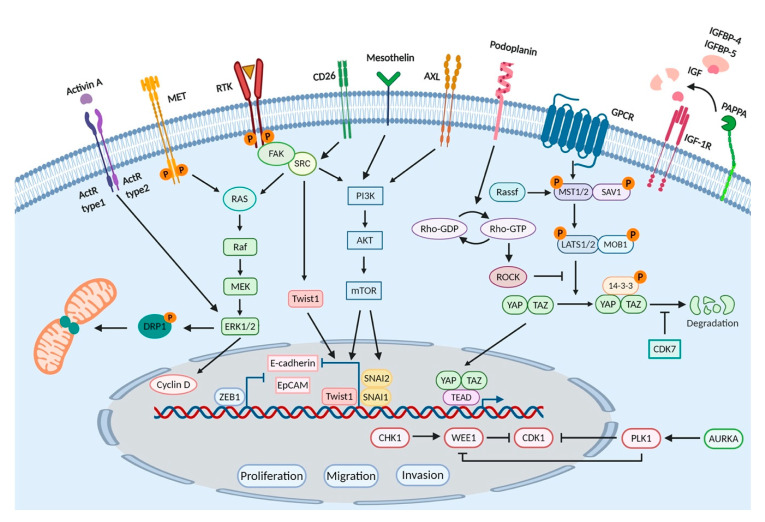
An outline of molecular mechanisms implicated in MPM pathogenesis through functional genomics studies.

Functional genomics aims to uncover the relationship between genetic aberrations and phenotypic characteristics of the disease, the ultimate goal of which is to translate the findings into clinical applications. The invention of a series of rapidly evolving large-scale genomic and transcriptomic sequencing technologies facilitated the detection and identification of novel mutations and gene expression deregulations in cancer. Indeed, by combining both “gene-by-gene”, which we refer to as low throughput here, and high-throughput studies with functional genomics tools, such as RNAi and CRISPR, researchers were able to execute the global characterization of complex relationships between the genotype and the phenotype. Even though the number of functional genomics studies in MPM is limited, the available literature successfully validates the functional aspects of numerous genetic alterations that are significantly associated with biological processes including transcription, signal transduction, cell division, and migration. Experimental models used in these studies include established cancer cell lines, next-generation cell culture systems, and animal models. In this section of the review, we summarize the MPM literature with a particular focus on functional genomics studies with diagnostic, prognostic, and therapeutic implications (Figure 1).

### 3.1. Low-Throughput Functional Studies

Historically, the “gene-by-gene” approach in low-throughput functional studies has been very common in cancer research. These studies have made remarkable contributions to the identification of certain cancer-associated genes/pathways with relevance to specific biological processes and disease states. Referring to them, here we provide a summary of RNAi-based loss of function studies in MPM.

Membrane proteins along with heterotypic signaling molecules are key players in intercellular communication and signal transduction. Based on their biological function, membrane proteins can be grouped as receptors, cell adhesion molecules, glycosylphosphatidylinositol (GPI)- and lipid-anchored proteins, transporters, and cell membrane-associated enzymes. Alterations in membrane proteins and, in some cases, their ligands have been associated with the progression of cancer [149]. In principle, all membrane proteins that are highly expressed in tumors have the potential to be utilized as diagnostic and prognostic biomarkers, and pharmaceutical targets. Receptor tyrosine kinases (RTKs) and the downstream molecules that convey signal transduction processes play crucial roles in cellular homeostasis and are amenable to deregulation in cancer. Indeed, mutations in the RTKs have been identified in several cancers [150], and an increasing body of evidence has emphasized their dysregulation in MPM [97,151,152]. In particular, the knockdown of known RTKs such as AXL, EPHB2 (Ephrin B2 receptor), and MET by RNAi (mainly shRNA) or inhibition by small-molecule inhibitors led to a marked reduction in cell viability and migration capacity [153,154,155]. Specifically, the AXL inhibitor was shown to block the interaction between the AXL and PI3K pathways resulting in the disruption of the PI3K/Akt/mTOR signaling, a rationale granting the evaluation of AXL inhibition as a therapeutic strategy against the clinical progression of MPM [153]. Moreover, Wang et al. has recently reported that silencing of MET expression via siRNA or pharmacological inhibition of its activity attenuated the viability and migration capacity of MPM cells in vitro and significantly impeded tumor growth in vivo. Mechanistically, this was explained by dephosphorylation of DRP1 (dynamin related protein), a key component of mitochondrial and peroxisomal division machinery, uncovering a novel mechanism of mitochondrial regulation by the crosstalk between DRP1 and MET pathways. Given these promising findings, further evaluation of combinatorial inhibition of MET signaling and DRP1 activity in MPM is warranted [155].

Similar to RTKs, receptor serine/threonine kinases (RSTKs), also known as activin receptors or activin-receptor-like kinases, are critical mediators of a wide array of cellular events such as proliferation, apoptosis, differentiation, and migration and play a role in the pathogenesis of many diseases, including cancer [156]. In MPM, Hoda et al. studied the dependency of MPM cell lines to activin receptor activity as well as the activins, members of TGF-β superfamily of secreted polypeptide growth factors. Their work showed that activin A was highly expressed in epithelioid phenotype and silencing of activin βA expression by siRNA attenuated cell viability and clonogenicity of MPM cell lines. Moreover, selective small molecule inhibition of type I activin receptors resulted in impaired growth, clonogenicity, and migration of MPM cell lines. Possible mechanisms underlying these observations included reduced expression of Cyclin D1 and Cyclin D3 and decreased phosphorylation of ERK1/2 [157]. Similarly, Tamminen et al. showed that all MPM samples tested in their study scored high immunoreactivity for activin A and activin B. Moreover, their independent work confirmed that ERK activation was required for activin A-induced motility and invasion of MPM cells [158]. Conclusively, individual or combinatorial inhibition of activins, activin-receptor-like kinases, and ERK pathway evokes a therapeutic window against MPM. 

CD26 is a type II transmembrane glycoprotein and is related with high proliferative activity as well as invasiveness in MPM cells. The knockdown of CD26 resulted in a significant reduction of periostin, a secreted cell adhesion protein. Similarly, p-Src levels and the expression of the transcription factor Twist, a regulator of periostin expression downstream of c-Src pathway, were significantly reduced upon CD26 silencing, revealing the mechanism of CD26-associated upregulation of periostin wherein increased secretion of periostin resulted in the enhanced migratory and invasive activity of MPM cells [159].

In the study by Ishiguro et al., activated leukocyte cell-adhesion molecule (ALCAM/CD166), which is also a cancer stem cell marker, has been shown to be highly expressed in MPM. Mechanistically, ALCAM enhanced the malignant features of mesothelioma cells and positively contributed to MPM progression. As exemplified by the efforts to study its function, silencing of ALCAM led to the inhibition of cell migration and invasion, and impaired anchorage-independent cell growth without affecting cell proliferation. Similarly, the administration of a purified and soluble form of ALCAM, namely, sALCAM, which is known to block homophilic binding of ALCAM, attenuated cell migration and invasion, and inhibited anchorage-independent cell growth in vitro. Similarly, subcutaneous tumors formed by the inoculation of the MPM cell line expressing sALCAM resulted in significantly prolonged survival in mice, offering therapeutic utility of this cell adhesion molecule in MPM [160].

Podoplanin, a well-conserved type-I transmembrane sialomucin-type glycoprotein encoded by the *PDPN* gene, is emerging as a candidate biomarker characterized by elevated expression in MPM. The knockdown of PDPN expression by siRNA or shRNA in cell lines expressing high levels of Podoplanin inhibited cell motility and resulted in decreased Rho-GTP binding. Reciprocally, ectopic expression of PDPN enhanced cell motility and resulted in increased Rho-GTP binding, evoking a motility promoting role for PDPN in MPM via the regulation of RhoA/ROCK pathway activity. Finally, a variety of modalities tailored to target both intracellular and extracellular domains of Podoplanin have demonstrated promising results in many cancers including MPM [161].

Mesothelin, a GPI-anchored plasma membrane differentiation antigen encoded by the *MSLN* gene, is often overexpressed in MPM patients. Because of its limited expression in normal tissues, mesothelin exhibits high specificity for malignancy. More importantly, mesothelin breaks down in the pleural cavity into smaller soluble proteins known as soluble mesothelin-related peptides (SMRPs) which can be detected in serum samples. These attributes granted mesothelin the Food and Drug Administration (FDA) approval for the diagnosis of MPM. Of particular interest, mesothelin is also being investigated as a therapeutic target. Recent work by He et al. has shown that the knockdown of MSLN expression by shRNA inhibited cell growth, colony formation, tumorsphere formation, migration, and invasion in vitro and reduced tumor formation and metastasis in vivo. Besides, the inactivation of mesothelin resulted in the upregulation and downregulation of several EMT markers (*CDH1* and *CAV2*) and stem cell specific genes (*TWIST*, *SNAI1*, *SNAI2*, and *ABCG2*), respectively, revealing its role in the regulation of the EMT process and cancer stem cell traits [162]. Further studies in a clinically relevant orthotopic mouse model that recapitulated the MPM biology showed that the ectopic overexpression of mesothelin promoted tumor invasion and significantly decreased survival, compared to mice with MSLN-negative tumors. This effect of mesothelin was attributed to increased expression and concomitant secretion and colocalization of MMPs, especially MMP-9, with mesothelin which substantiated the biological role of mesothelin as a promoter of tumor invasion and highlighted the translational relevance of these findings [163].

To date, several studies have shown that the activation of the Hippo pathway and the YAP protein are important in MPM, and enhanced oncogenic activity of YAP has been associated with poor prognosis. The inhibition of YAP by siRNAs or Verteporfin, a YAP-specific inhibitor, significantly suppressed invasion and tumorsphere formation of MPM cells by repressing downstream gene transcription of the Hippo kinase cascade, suggesting that MPMswith aberrant YAP activity could be sensitive to Verteporfin therapy [164]. In addition, the inactivation of RhoA/ROCK signaling by siRNAs or GSK269962A, a selective ROCK inhibitor, decreased YAP transcriptional activity and the viability of MPM cells [164]. Furthermore, CDK7 (cyclin-dependent kinase 7), a serine/threonine protein kinase, can sustain Hippo signaling by directly phosphorylating and protecting YAP from ubiquitination and degradation [165]. In a study by Mioa et al., the inhibition of CDK7 by siRNAs downregulated YAP expression via promoting its degradation and suppressed the invasion and tumorsphere formation capacity of MPM cells [166]. As their druggability potential has been assessed positively in other cancer types, the results of these studies suggest that RhoA/ROCK pathway and CDK7 represent therapeutically actionable targets in MPM as well.

MPM is certainly an aggressive cancer characterized by increased migration and subsequent invasion and metastasis into other tissues and organs. In an attempt to identify the genes that are related to MPM cell migration, Huang et al. investigated the migration capacity and gene expression profiles of MPM cell lines and determined that several genes have had relatively high correlation with migration. For example, the increased expression of pappalysin 1, a secreted metalloprotease encoded by the *PAPPA* gene which selectively cleaves insulin-like growth factor binding proteins (IGFBP-4 and IGFBP-5), positively correlated with the augmented migratory capacity of MPM cell lines. As expected, the enhanced cellular migration was associated with the proteolysis of IGFBP-4 by pappalysin 1 and the subsequent release of IGF-1 as a chemotactic factor. Furthermore, siRNA- or shRNA-mediated silencing of PAPPA led to inhibition of migration and proliferation of MPM cells and inhibited tumor growth in mouse orthotopic xenograft models, suggesting that pappalysin 1 could represent a potential therapeutic target [167].

Transcription factors are generally deregulated in carcinogenesis and are potential targets for cancer therapy [168]. Functional consequences of deregulations in ZEB1, ERβ, SP1, and WT1 zinc-finger transcription factors are well studied in breast cancer, lung cancer, pancreatic cancer, and prostate cancer [169,170,171,172]. Recently, the upregulation of these genes has been shown in MPM, when compared to matched normal counterparts [173,174,175,176]. Horio et al. showed that siRNA-mediated knockdown of ZEB1 (zinc finger e-box binding homeobox 1) led to the suppression of proliferation, and anchorage-dependent and anchorage-independent clonal growth of MPM cells. Besides, the stable knockdown with a retroviral shRNA-expressing vector resulted in transcriptional derepression of E-cadherin and EpCAM, thus mesenchymal-to-epithelial (MET) transition. The findings of this study suggest that ZEB1 may serve as a promising therapeutic target in MPM [177]. In line with this, the knockdown of estrogen receptor beta (ERβ) in ER-positive REN cell line, an MPM cell line with epithelioid histotype, resulted in increased EGFR, Akt, and ERK1/2 phosphorylation, and promoted proliferation. Additionally, ERβ colocalized with EGFR in Caveolin 1-enriched membrane fractions leading to delayed internalization of ERβ by interfering with EGFR phosphorylation. Suggestive of a therapeutic role, ERβ silencing sensitized REN cells to Gefitinib treatment [178]. In the study by Rao et al., shRNA-mediated knockdown of SP1 (Sp1 transcription factor) inhibited the growth, migration, and tumorigenicity of MPM cells, imposing an oncogenic function for SP1 in MPM. Additionally, treatment with mithramycin (MM), an anti-neoplastic agent that inhibits binding of SP1 to DNA, decreased SP1 expression and inhibited the proliferation and clonogenicity of MPM cells in vitro, and tumorigenicity in vivo [175]. The knockdown of WT1 (Wilms Tumor Protein 1), another transcription factor highly expressed in MPM, greatly reduced the proliferation, chemotaxis, and invasion of MPM cells. Although not significantly, WT1 knockdown improved chemosensitivity to cisplatin treatment, granting the necessity for additional studies [179].

Pemetrexed is an anticancer chemotherapy drug used in the treatment of MPM. Unfortunately, about half of the tumors are inherently chemoresistant, limiting the wide application of that antifolate small molecule. The resistance to pemetrexed has been largely attributed to the increased expression of the thymidylate synthase (TS) enzyme, a folate-dependent enzyme responsible for the conversion of deoxyuridine monophosphate (dUMP) to deoxythymidine monophosphate (dTMP), a rate-limiting step in DNA synthesis. Abu Lila et al. investigated the effect of the downregulation of *TS* gene on the cytotoxic efficacy of pemetrexed against MPM. Combined treatment of human MPM cell line representing biphasic subtype, MSTO-211H, with TS-specific shRNA and pemetrexed resulted in a remarkably higher cytotoxicity when compared to pemetrexed alone. Similar results were obtained in vivo where doublet combination treatment of orthotopic MSTO-211H tumors, implanted in the thoracic cavity of nude mice, with TS shRNA and pemetrexed resulted in marked inhibition of tumor burden and improved overall survival [180]. In support of these findings, Monica et al. showed that dasatinib, a dual Bcr/Abl and SFK (the Src family of protein tyrosine kinases) competitive ATP inhibitor, sensitized MPM cell lines to pemetrexed treatment by reducing TS protein expression. Thus, the use of effective antifolate thymidylate synthase inhibitors in combination with pemetrexed regimens could represent a viable therapy option in MPM treatment [181].

### 3.2. High-Throughput Functional Genomics Screens

Often combined with a wide array of high throughput technologies, such as genome-wide RNAi and CRISPR screens as well as drug screening, functional cancer genomics studies entail the functional annotation of cancer-causing aberrations.

#### 3.2.1. RNA Interference and CRISPR Screens

The study by Sudo et al. marks the first large-scale loss of function RNAi screen conducted in MPM cells. The siRNA library used in this work consisted of nine sub-libraries, namely, ion binding, ion channel, kinase, membrane transporter, nucleic acid binding, phosphatase, receptor, transcription factor, and transporter, culminating to 8589 siRNAs in total. The study found that the knockdown of 78 genes caused suppression in the proliferation of MSTO-211H cell line by at least 80%. These genes were further validated in a secondary sub-library screen where *COPA*, *COPB2*, *EIF3D*, *POLR2A*, *PSMA6*, *RBM8A*, and *RPL18A* genes showed the greatest negative impact on cell viability. The authors then focused on *COPA*, a gene that encodes a subunit of the cargo complex that modulates retrograde protein trafficking from Golgi to the endoplasmic reticulum, for functional studies in vivo as it had been reported to have increased expression in MPM. Functional studies with MSTO-211H cell line revealed that the knockdown of COPA gene by siRNA suppressed tumor growth and induced apoptosis in vivo, suggesting that COPA could be a novel therapeutic target in MPM [182].

To identify novel genes with MPM-specific therapeutic relevance, Linton et al. selected 40 genes for functional studies. These genes were previously reported to be overexpressed in MPM [183,184]. In particular, the silencing of *BIRC5*, *CDK1*, *CHEK1*, *NDC80*, *PLK1*, *RRM1*, or *RRM2* genes resulted in a profound reduction in cellular growth. The authors further evaluated the role of CDK1 (cyclin dependent kinase 1), NDC80 (kinetochore complex component), and PLK1 (polo like kinase 1) proteins in cell survival since they could be targeted by small-molecule inhibitors and have not been linked to MPM before. Indeed, treatment of a panel of human MPM cell lines (H28, MSTO-211H, MM05, and H226) with selective CDK1, NDC80, or PLK1 small-molecule inhibitors attenuated cell growth and colony formation. Additionally, small-molecule inhibitors of CDK1 and NDC80 increased cisplatin sensitivity of MPM cells [185]. In conclusion, this study showed that RNAi screens could be harnessed for the functional interrogation of genes on a large scale and help uncover new genes and signaling pathways with therapeutic implications in MPM.

The only frontline chemotherapy regimen approved for clinical use in advanced stage MPM is the combination of platin and pemetrexed. Unfortunately, only a small fraction of patients responds to this therapy and inevitably most cases develop drug resistance. A recent functional genomics study by Xu et al. has aimed to identify the genetic determinants that limit the efficacy of standard cisplatin/pemetrexed chemotherapy in MESO-1 cells by implementing a kinome-wide CRISPR negative selection screen strategy based on a lentiviral sgRNA library that targets 763 kinases. The screen revealed that 33 kinases were markedly depleted in cisplatin/pemetrexed-treated versus the vehicle control group. Six kinase genes, namely, *WEE1*, *AURKA*, *MPP3*, *MAP3K12*, *DGKD*, and *SPEG*, scored for the most significant screening criteria. Of note, the depletion of WEE1, a G2-M checkpoint kinase with therapeutic implications, showed the most remarkable negative effect on cell viability. Accordingly, the authors performed follow up experiments on *WEE1* gene and demonstrated that CRISPR-based deletion of *WEE1* sensitized MPM cells to cisplatin/pemetrexed treatment. More importantly, inhibition of WEE1 by a small-molecule inhibitor AZD1775 showed synergistic effects with chemotherapy agents by forcing a premature mitotic entry and led to enhanced MPM cell death in vitro, which was further harmonized by potent antitumor effects in vivo [186].

Very recently, Okonska et al. have performed a genome-wide synthetic lethality siRNA screen in H2452 cell line that was engineered to express either functional or nonfunctional BAP1. Thus, the aim was to define BAP1-dependent genetic vulnerabilities. The study identified 1775 genes, among which 191 genes were calculated to be significantly differentially lethal, when lethality score was set to ≥0.2 between the BAP1-proficient vs BAP1-deficient cells. Interestingly, gene ontology analysis revealed that the most significantly enriched functional annotation group of these 191 genes was RNA splicing and processing. Furthermore, using a more stringent differential lethality score, the authors identified 11 genes whose depletion were specifically more cytotoxic in the BAP-proficient cells. From this cluster of 11 genes, *RRM1* (ribonucleotide reductase catalytic subunit M1) and *RRM2* (ribonucleotide reductase catalytic subunit M2) genes were selected for further characterization as they were deemed to have clinical relevance. Specifically, RRM1 and RRM2 subunits form the ribonucleotide reductase (RNR) protein heterotetramer complex, a key enzyme in dNTP synthesis that helps maintain DNA replication fidelity and genome stability. Noteworthy, the inhibition of elevated RNR activity by gemcitabine, alone or in combination with other drugs, has been exploited for second line therapy in MPM for many years. By measuring the sensitivity of a set of MPM cell lines to RRM1 and RRM2 inhibition both in two dimensional (2D) and three dimensional (3D) cell culture conditions, the authors found that BAP1-proficient cell lines (*BAP1* wild type: H2052, ACC-Meso-1, Mero82, and SPC111) were more sensitive to this challenge, when compared to BAP-deficient cell lines (*BAP1* mut/del: ACC-Meso-4, H226, and H2452). Similarly, the loss of function of BAP1 resulted in chemoresistance to RNR inhibition, suggesting that the BAP1 status may define a synthetically lethal genetic vulnerability, and could be a predictor of therapy response [187].

High-throughput RNAi and CRISPR screens provide a tremendous opportunity for the systematic characterization of cancer dependencies. However, they can be technically challenging to implement. While many high-throughput screens have been conducted in various human cancer cell lines, as outlined above, such studies are limited in MPM. Nevertheless, several resources are now available for public use which can help cancer researchers identify unique vulnerabilities and cancer-specific dependencies. One such resource is The Cancer Dependency Map (DepMap) portal [188,189], which was established through a collaboration between Broad Institute and Welcome Sanger Institute. With a commitment to Open Science, the DepMap, an ongoing project receiving frequent updates, provides free access to a wide range of information for more than 700 established cancer cell lines, including several MPM cell lines. Large datasets featured in the DepMap portal include cell line-specific genetic and transcriptomic profiles, sensitivity to small molecule perturbations, and genome-wide RNAi and CRISPR loss of function screens. To demonstrate the feasibility of this resource, here we performed a proof of principle analysis on select MPM cell lines. Using CRISPR (Avana) Public 20Q1 [190] and Combined RNAi datasets [191], we analyzed the common vulnerabilities of five MPM cell lines: H2052, H2452, H28, ACC-MESO-1, and MPP89. For this analysis, we defined the cut-off values as −1.5 and −0.75 for CRISPR and RNAi screen datasets, respectively. Following a manual curation, we found that among the genes with significant depletion scores *DDB1*, *EEF2*, *EIF3A*, *HSPE1*, *RAN*, *RPS15A*, *RUVBL1*, *SNRPD1*, *UBA1*, and *VCP* genes were common in both datasets and all five cell lines. Encouragingly, all the genes obtained in this analysis have been previously listed as common essential genes [192], see Table 3. 

**Figure 2:**
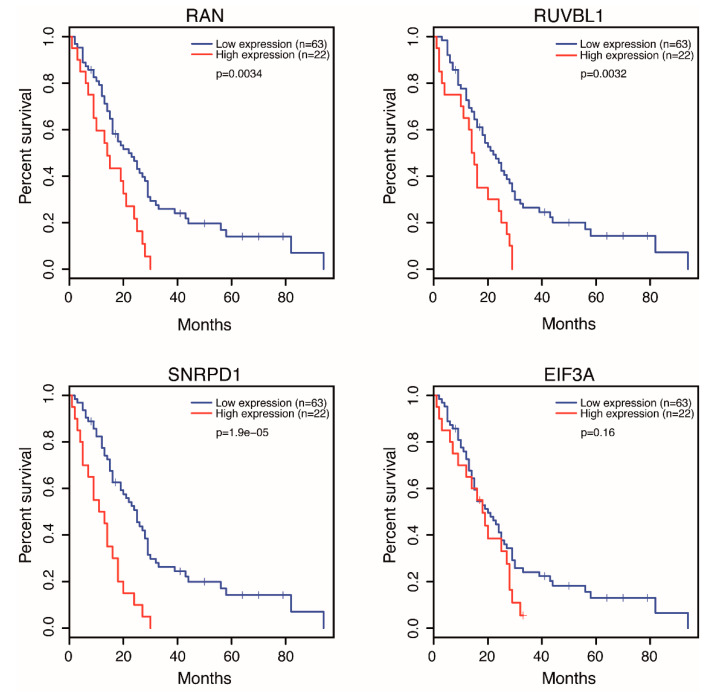
Overall survival rates of MPM patients according to low and high expression levels of select genes.

We further extended the analysis and investigated the mutational status of these genes in the TCGA dataset of 87 MPM cases. We found that *DDB1* and *EEF2* genes were amplified in two and one cases, respectively. Similarly, we determined that *EIF3A* and *RUVBL1* genes were deleted in two individual cases suggesting that the genetic aberrations in these genes are infrequent. We hypothesized that for some, if not all, genes high dependency scores could be explained, in part, by gene expression alterations and overall survival profiles of MPM patients. To test this, we took the advantage of the intuitive, user-friendly interface of the GEPIA [193] and the UALCAN [194] web portals. This analysis revealed that the high expression levels of *RAN*, *RUVBL1*, and *SNRPD1* genes were significantly associated with lower overall survival rates (Figure 2).

More importantly, among these 10 genes, the *RAN* gene, which encodes a small RAS superfamily GTP binding protein that is essential for a variety of nuclear processes, was highly expressed in and was proposed as a drug co-target against MPM [183]. A very recent study by Dell’Anno et al. also showed that siRNA-mediated depletion of RAN expression in MPM cell lines abrogated cellular growth and colony formation, suggesting that RAN signaling acts as a positive regulator of survival and proliferation in MPM [195]. Furthermore, the high expression levels of *EIF3A* gene seemed to correlate, although not significantly, with lower overall survival rates (Figure 1). Plus, an RNAi screen by Sudo H et al. showed that the knockdown of EIF3A reduced the viability of MPM cells, suggesting that further studies preferably on larger MPM cohorts, would be necessary to define a statistically significant correlation, if existing, between EIF3A expression and the overall survival of MPM patients [182]. In addition, the DepMap portal can also provide a top 10 preferentially essential genes list for each screened cell line. Unlike our manually curated gene list, that list is computed in a cell line-specific manner by subtracting the mean score of a specific gene across all cell lines, calculating the lowest mean-subtracted score of that gene for a given cell line. In conclusion, executing data curation based on different selection criteria can effectively provide scientists with distinct results that would complement their research interests.

As illustrated, a straightforward and systematic proof of principle approach based on the sequential use of various resources and computational tools enabled us to define a list of 10 common genes in CRISPR and RNAi screen datasets across all five cell lines. Among these, four genes were associated with MPM survival and two genes were previously linked to MPM, revealing that high throughput data presented through these portals can provide reliable sources for hypothesis development and testing, as well as generating preliminary data for functional studies. By leveraging other features of the DepMap project, researchers can further unveil the landscape of cancer vulnerabilities for therapeutic development and even identify biomarkers that predict them.

#### 3.2.2. Drug Screens

MPM is a heterogeneous cancer with poor druggable biological targets. With an effort to study the efficacy of new drug candidates and regimens, researchers have employed high-throughput methods and tools in recent years. In a study by Quispel-Janssen et al., the researchers utilized a high throughput drug screen with 265 different chemical inhibitors in a panel of 889 cancer cell lines, including 19 MPM cell lines. Three of those MPM cell lines showed very high sensitivity to PD-173074, an FGFR1 and FGFR3 kinase inhibitor (FGFRi). This sensitivity was further confirmed by other more selective FGFR inhibitors, NVP-BGJ398, and AZD4547. Gene expression analysis of FGFRi-sensitive and FGFRi-resistant MPM cell lines revealed a remarkable correlation between elevated FGF9 mRNA expression and treatment response to PD-173074 and AZD4547. Notably, there was a significant correlation between loss of BAP1 expression, but not mutation status, and AZD4547 sensitivity. Further, AZD4547 treatment resulted in a significant growth inhibition in the FGFRi-sensitive MPM lines that were reconstituted from tumors, confirming the therapeutic potential of the FGFR pathway in a subset of MPM patients. Finally, the cell lines not responsive to FGFR inhibition were found synergistically sensitive to IGF1R and PI3K inhibition, suggesting that intrinsic resistance to FGFRi could be overcome by combinatorial therapies [196]. In a follow up study, the same group performed a large-scale drug screen consisting of seven common compounds (cisplatin, carboplatin, oxaliplatin, vinorelbine, gemcitabine, pemetrexed, and doxorubicin) and their combinations (cisplatin + pemetrexed, cisplatin + gemcitabine, carboplatin + pemetrexed, oxaliplatin + gemcitabine, and oxaliplatin + vinorelbine) on a set of 81 different primary cultures. Based on drug response rates, cultures were clustered into clinically relevant subgroups as the responders, nonresponders, and intermediate responders. To study the genomic basis of differential drug responses, RNA sequencing was performed. FGF9 and its receptors FGFR1 and FGFR3 were found to be upregulated in the nonresponders, in comparison to the responders. Correspondingly, the nonresponder primary cultures were found to be sensitive to the FGFR inhibitor, PD-173074. More importantly, in vitro drug responses of primary cultures were predictive of clinical responses to select drugs, suggesting that preliminary culture systems are suitable to identify mechanistic determinants of drug responses, and personalize treatment regimens [197].

## 4. In Vitro and In Vivo Model Systems in Malignant Pleural Mesothelioma Research

In the current MPM research, established and commercially available cell lines are frequently used in functional genomics studies. In general, 2D cell culture systems and the subcutaneous, and orthotopic xenograft models derived from them are suitable for the identification and functional validation of cellular, molecular, and genetic mechanisms of the disease, and the initial evaluation of potential antitumor agents. Yet there are various limitations associated with them. In particular, these models fail to completely recapitulate the genetic and phenotypic heterogeneity of the human disease, primarily the inflammatory nature, and intra- and inter-tumor heterogeneity of MPMs. Today, there is a growing interest in developing new experimental models for MPM studies, in particular in vitro next generation 3D cell culture systems such as organoids, in vivo preclinical tumor models such as patient-derived xenografts (PDX), and of course, genetically engineered mouse models (GEM) that can collectively facilitate the low and high throughput functional validation studies of genetic perturbations, assessment of drug responses, and identification of druggable targets [198,199,200,201,202,203,204].

Three dimensional MPM models provide many advantages over 2D cultures as they better represent the major in vivo characteristics of tumors. In a recent report, Mazzocchi et al. have collected surgical biospecimens from two human MPM patient tumors and generated a series of patient-derived organoids that accurately represented the disease and tissue heterogeneity. Furthermore, when integrated into a microfluidic organ-on-a-chip system to better mimic in vivo conditions, these MPM organoids were successfully used as a means to discover, with high clinical significance, patient-specific drug sensitivities, and predictive biomarkers against small molecules, demonstrating the remarkable capability of this platform to personalize and optimize treatment regimens [198]. The PDX models involve the collection of specimens from patient tumors and the successive subcutaneous implantation of these tissues or cells into immunocompromised or humanized mice. Notably, the PDX models retain the main pathohistological and genetic features of original tumor tissues and the associated stroma, to a lesser degree. A few years ago, Wu et al. attempted to establish the first large collection of PDX models from all histological subtypes. A total of 50 tumor samples were subcutaneously inoculated into flanks of NOD/SCID mice. Twenty of these were engrafted and passaged successfully for multiple generations. Further evaluation revealed that the PDX models effectively reflected the clinical heterogeneity of the respective parent tumors, and responded to Cisplatin treatment, proving the utility of these models to identify predictive biomarkers and evaluate the efficacy of treatment regimens [199].

As noted previously, oncogenic gain of function alterations seldom occurs in MPM. Rather, MPM is characterized by frequent genetic losses of *CDKN2A*, *NF2*, *BAP1*, and *TP53* tumor suppressor genes. Accordingly, most of the MPM-specific GEM models, if not all, developed to date have focused on introducing these specific genetic aberrations of the human disease. To exemplify, Altomare et al. developed a model where repeated asbestos treatment of heterozygous *NF2* mice induced rapid onset of MPM. Consistent with human malignancy, the tumors recapitulated the genetic and molecular features of MPM, characterized by the inactivation of the normal allele of the *NF2* gene as well as frequent homozygous co-deletions of the *CDKN2A* and *CDKN2B* genes and the activation of Akt signaling. Collectively, this model is found to be practical for testing novel therapeutic strategies against growth and survival signaling pathways (e.g., the Akt pathway) implicated in this deadly disease [200].

Later, using a CRE-LoxP system by direct injection of Adenovirus expressing Cre recombinase (Adeno-Cre) into the pleural cavity of adult mice, Jongsma et al. developed compound mouse models carrying the combinations of conditional knockout alleles for *NF2* and *INK4A* and/or *TP53* that developed tumors with varying tumor latency, aggressiveness, and survival rates. As clearly stated by the investigators, this murine model may provide a mechanistic framework for the discovery of additional recurrent genetic alterations and unique gene expression deregulations. Besides, this model can help to explore novel molecular cues associated with MPM development and eventually enable the design of improved therapeutic strategies [201].

Recently, Sementino et al. have generated animal models to study the cooperation between *PTEN* and *TP53* inactivation in MPM pathogenesis. Their study successfully demonstrated that the compound inactivation of *PTEN* and *TP53* genes in mouse mesothelium, by intrathoracic injection of Adeno-Cre, could synergistically drive highly penetrant, rapid, and aggressive tumors, even in the absence of exposure to asbestos. Notably, this model can have translational implications because the *TP53* gene is mutated in ~15% of human MPMs and *CDKN2A* deletions, therefore the functional loss of the p14ARF protein, can also result in the deregulation of the p53 pathway. Moreover, the PI3K/Akt/mTOR pathway is activated in most human MPMs. Indeed, the tumors and the cell lines derived from this model were mainly characterized by genomic instability, upregulation of Myc, and hyperactivation of PI3K/Akt/mTor signaling. Although not feasible for testing therapeutic strategies aiming to reactivate TP53 and PTEN pathways, the rapid onset and short latency period of these tumor models and the frequent deregulation of therapeutically viable signaling pathways make this GEM model suitable for preclinical applications [202].

As shortly discussed earlier, individuals carrying germline mutations in the *BAP1* gene are predisposed to MPM. Independent studies conducted in knockout animal models harboring germline heterozygous mutations (*Bap1*+/−) showed that these models could develop spontaneous tumors with low incidence. Furthermore, asbestos exposure of *Bap1*+/− animals resulted in accelerated, larger, and more aggressive tumor development with very high incidence and low survival rates. These findings were further supported by the accumulation of additional genetic aberrations such as the loss of the WT allele of the *BAP1* gene and homozygous deletions of the *CDKN2A* and *CDKN2B* genes, recapitulating the features of human MPM pathology. The studies in these models proved that the *BAP1* gene was a bona fide tumor suppressor whose heterozygous germline mutation increases the risk of MPM development in *BAP1* mutation carriers when exposed to environmental carcinogens, in particular asbestos fibers [205,206,207].

To understand the tumorigenic potential of the most frequently altered genetic lesions in MPM development, Badhai et al. have recently developed mouse models based on single and compound inactivation of tumor suppressor genes, namely *BAP1*, *NF2*, and *CDKN2A* and *CDKN2B* (*CDKN2AB*). In short, only the compound models *BAP1*, *NF2*, and *CDKN2AB* (dubbed as BNC, combined deletion of *BAP1*, *NF2*, and *CDKN2AB*), and *NF2* and *CDKN2AB* (dubbed as NC, combined deletion of *NF2* and *CDKN2AB*) developed highly aggressive tumors that recapitulated the histological and molecular characteristics of human patients, such as inflammatory microenvironment and gene expression profiles. More importantly, the BNC autochthonous tumors responded to and developed resistance against frontline chemotherapy agents with similar dynamics in human tumors. Similarly, the BNC tumors exhibited concurrent activation of MAPK and PI3K pathways and cell lines derived from these tumors were sensitive to PI3K inhibitor BEZ-235 [203]. Taken together, this model may be suitable for testing new treatment modalities including new immunotherapy concepts, screening synthetic lethal interactions, and investigating resistance mechanisms.

In another study by Kukuyan et al., the researchers created conditional homozygous knockout models of *BAP1*, *NF2*, and *CDKN2A* tumor suppressor genes based on intrathoracic injection of Adeno-Cre. Specifically, triple knockout animals developed highly invasive and proliferative tumors, predominantly with sarcomatoid histologic subtype, recapitulating the human MPMs. RNA sequencing analysis of triple knockout tumors displayed increased expression of genes previously implicated in BAP1 function. More importantly, when compared to *NF2* and *CDKN2A* double knockout tumors where BAP1 expression was retained, many of the top differentially expressed genes in triple knockout tumors were downstream targets of PRC2, uncovering a connection between BAP1 and PRC2 and thereby confirming in vivo how, at least in part, the loss of BAP1 contributes to MPM pathogenesis [204].

## 5. Conclusions and Future Challenges

Clinical trials evaluating the efficacy of biologic agents that target key pathways and recurrent alterations in MPM failed to provide the expected results. A possible explanation to this failure may lie in the lack of oncogenic driver mutations in MPM, which instead characterize other types of cancer. Rather, the loss of tumor suppressor genes results in the simultaneous dysregulation of different downstream pathways. For example, the loss of Merlin activity triggers cell proliferation through Hippo, PI3K/Akt/mTOR, and FAK pathways. In that regard, targeting transduction pathways individually has been ineffective in abrogating the proliferative potential of cancer cells. These negative results highlight the necessity to better understand the MPM pathobiology. Accordingly, a substantial number of basic and translational research efforts, covering the comprehensive evaluation of cellular and molecular features and their interconnections and relationships with the tumor microenvironment, are already in progress. The primary aim is to identify new risk factors and novel therapeutic targets and treatment strategies in MPM, and integrative and high throughput genomics and functional genomics studies can definitely help advance these efforts.

## Figures and Tables

**Table 1:** Genomic analysis of the TCGA malignant pleural mesothelioma (MPM) cohort using the cBioPortal Resource. N/A: data not available.

Gene Symbol	Gene Name	Chromosomal Location	GO Annotation	Molecular Function	Mutation Frequency (%)	CNA Frequency (%)	CNA Type	Fusion Frequency (%)
NF2	Neurofibromin 2	22q12.2	Actin binding and cytoskeletal protein binding	Interacts with cell-surface proteins, proteins involved in cytoskeletal dynamics and proteins involved in regulating ion transport. *Tumor suppressor*	23.3	8.0	HOMDEL	2.3
BAP1	BRCA1 Associated Protein 1	3p21.1	Chromatin binding and thiol-dependent ubiquitin-specific protease activity	Binds to the breast cancer type 1 susceptibility protein (BRCA1) via the RING finger domain of the latter and acts as a tumor suppressor. *Tumor suppressor*	20.9	11.5	HOMDEL	3.5
TP53	Tumor Protein P53	17p13.1	DNA-binding transcription factor activity and protein heterodimerization activity	Acts as a tumor suppressor in many tumor types; induces growth arrest or apoptosis depending on the physiological circumstances and cell type. *Tumor suppressor*	16.3	N/A	N/A	N/A
TTN	Titin	2q31.2	Nucleic acid binding and identical protein binding	Key component in the assembly and functioning of vertebrate striated muscles	14.0	N/A	N/A	N/A
LATS2	Large Tumor Suppressor Kinase 2	13q12.11	Transferase activity, transferring phosphorus-containing groups and protein tyrosine kinase activity	Negative regulator of YAP1 in the Hippo signaling pathway that plays a pivotal role in organ size control and tumor suppression by restricting proliferation and promoting apoptosis. *Tumor suppressor*	9.3	1.1	HOMDEL	N/A
SETD2	SET domain containing 2, histone lysine methyltransferase	3p21.31	Histone-lysine *N*-methyltransferase activity	Histone methyltransferase that specifically trimethylates H3K36me3 using H3K36me2 as substrate. *Tumor suppressor*	9.3	3.4	HOMDEL	3.5
PTCH1	Patched 1	9q22.32	Cholesterol binding	Acts as a receptor for secreted hedgehog ligands. *Tumor suppressor*	4.7	N/A	N/A	N/A
FAT4	FAT Atypical Cadherin 4	4q28.1	Calcium ion binding	Plays a role in the maintenance of planar cell polarity as well as in inhibition of YAP1-mediated neuroprogenitor cell proliferation and differentiation. *Tumor suppressor*	4.7	1.1	HOMDEL	N/A
FLG	Filaggrin	1q21.3	Calcium ion binding and structural molecule activity.	Aggregates keratin intermediate filaments and promotes disulfide-bond formation among the intermediate filaments during terminal differentiation of mammalian epidermis.	3.5	1.1	AMP	N/A
LRP4	LDL Receptor Related Protein 4	11p11.2	Calcium ion binding and scaffold protein binding	Functions as a specific facilitator of SOST-mediated inhibition of Wnt signaling.	3.5	N/A	N/A	N/A

**Table 2:** Dysregulated ncRNAs with biological activity in MPM. N/A: data not available.

Name of the ncRNA	Sample Type	Deregulation	Value	Functions	References
miR-98, miR-1271, miR-128, miR-143, miR-28, miR-3607, miR-500a, miR-532	Tumor Samples	Downregulation	Prognostic	These miRNAs are downregulated in asbestos-exposed group compared to non-exposed MPM patients. High expression of only miR-98 was significantly associated with poor prognosis in patients with asbestos-exposed MPM.	[143]
miR-200c, miR-210, miR-143	Pleural Effusions	Upregulation/downregulation	Biomarker	Upregulation of miR-210 and downregulation of miR-200c and miR-143 are potential PE cell biomarkers for differentiating MPM from benign and malignant PE-causing diseases.	[144]
miR-566	Cell Lines	High expression	Prognostic	Increased miR-566 expression was significantly associated with higher MT protein level which is related with drug resistance.	[140]
miR-548a-3p, miR-20a	Serum	High expression	Biomarker	Sera miRNA-548a-3p and miR-20a are elevated in MPM patients compared with cancer-free controls.	[145]
miR-15a, miR-15b, miR-16	Cell Lines	Low expression	N/A	Loss of the miR-15/16 family plays a role in the overexpression of the FGF axis in MPM. Restoration of miR-15/16 led to dose-dependent growth inhibition in MPM cell lines.	[146]
miR-137	Tumor Samples and Cell Lines	Variable expression—mostly high expression	Prognostic	High miR-137 expression is associated with poor patient outcome. Increased miR-137 levels by mimic transfection suppressed MPM cell growth and colony formation and reduced invasion by targeting YBX1.	[147]
miR-2053	Serum	High expression	Prognostic	Kaplan and Meier analysis revealed a significant increase in PFS (progression free survival) and decrease in cumulative hazards among miR-2053 positive MPM patients.	[148]
lncRNA-RP1-86D1.3	Serum	High expression	N/A	Had higher expression in MPM compared to benign and healthy control group.	[148]

**Table 3:** Genomic and functional details on the common essential genes in MPM cell lines.

Gene Symbol	Gene Name	Chromosomal Location	GO Annotation	Functions	Pathway/Superfamily
DDB1	Damage Specific DNA Binding Protein 1	11q12.2	Nucleic acid binding and damaged DNA binding	Binds to DNA following UV damage and functions in nucleotide-excision repair	DDB1 gene family
EEF2	Eukaryotic Translation Elongation Factor 2	19p13.3	Protein kinase binding	Catalyzes the GTP-dependent ribosomal translocation step during translation elongation.	GTP-binding translation elongation factor family
EIF3A	Eukaryotic Translation Initiation Factor 3 Subunit A	10q26.11	Translation initiation factor activity	Functions in the initiation of protein synthesis	eIF-3 subunit A family
HSPE1	Heat Shock Protein Family E (Hsp10) Member 1	2q33.1	Chaperone binding	Facilitates the correct folding of imported proteins together with Hsp60	GroES chaperonin family
RAN	RAN, Member RAS Oncogene Family	12q24.33	GTP binding	GTPase involved in nucleocytoplasmic transport	RAS superfamily
RPS15A	Ribosomal Protein S15a	16p12.3	Structural constituent of ribosome	Structural component of the ribosome. Oncogene	Universal ribosomal protein uS8 family
RUVBL1	RuvB Like AAA ATPase 1	3q21.3	ATPase activity and DNA helicase activity	Possesses single-stranded DNA-stimulated ATPase and ATP-dependent DNA helicase (3’ to 5’) activity	ATPases associated with diverse cellular activities (AAA+) protein family
SNRPD1	Small Nuclear Ribonucleoprotein D1 Polypeptide	18q11.2	RNA binding	Plays role in pre-mRNA splicing as core component of the SMN-Sm complex and the spliceosomal U1, U2, U4 and U5 snRNPs	SNRNP core protein family
UBA1	Ubiquitin Like Modifier Activating Enzyme 1	Xp11.3	Ubiquitin-like modifier activating enzyme activity	Catalyzes the first step in ubiquitin conjugation to mark cellular proteins for degradation through the ubiquitin–proteasome system	Ubiquitin-activating E1 family
VCP	Valosin Containing Protein	9p13.3	Signaling receptor binding	Necessary for the fragmentation of Golgi stacks during mitosis and for their reassembly after mitosis	AAA ATPase family

## Abbreviations

2D	Two dimensional
3D	Three dimensional
Adeno-Cre	Adenovirus expressing Cre recombinase
ALCAM	Activated leukocyte cell-adhesion molecule
BAP1	BRCA1-associated protein 1
BARD1	BRCA1-associated RING domain protein 1
BNC	BAP1, NF2, and CDKN2AB
BRCA1	Breast Cancer Type 1
BUB1	BUB1 mitotic checkpoint serine/threonine-protein kinase
cBioPortal	The cBio Cancer Genomics Portal
CD79B	CD79b molecule
CDK1	Cyclin dependent kinase 1
CDK7	Cyclin-dependent kinase 7
CDKI	Cyclin-dependent kinase inhibitors
CDKN2A	Cyclin-dependent kinase inhibitor 2A
CDKN2B	Cyclin-dependent kinase inhibitor 2B
CGH	Comparative genomic hybridization
CNA	Copy number alterations
COSMIC	Catalogue of Somatic Mutations in Cancer
c-Src	SRC proto-oncogene, non-receptor tyrosine kinase
DDB1	Damage specific DNA binding protein 1
DepMap	The Cancer Dependency Map
DRP1	Dynamin related protein
dTMP	Deoxythymidine monophosphate
dUMP	Deoxyuridine monophosphate
ECM	Extracellular matrix
EEF2	Eukaryotic translation elongation factor 2
EGFR	Epidermal growth factor receptor
EGFR-TKI	EGFR tyrosine kinase inhibitor
EIF3A	Eukaryotic translation initiation factor 3 subunit A
EIF3A	Eukaryotic translation initiation factor 3 subunit A
EMT	Epithelial-mesenchymal transition
EPHB2	Ephrin B2 receptor
ERM	Ezrin, Radixin, and Moesin
ERβ	Estrogen receptor beta
EZH2	Enhancer of zeste homolog 2
FAK	Focal adhesion kinase
FAT4	FAT Atypical Cadherin 4
FDA	Food and Drug Administration
FGF	Fibroblast growth factor
FGFRi	FGFR kinase inhibitor
FLG	Filaggrin
GEM	Genetically engineered mouse
GEPIA	Gene expression profiling interactive analysis
GPI	Glycosylphosphatidylinositol
H3K27me3	H3 lysine methylation
HCF-1	Host cell factor 1
HIF-1α	Hypoxia-inducible factor 1α
Hippo	Salvador-Warts-Hippo
HSPE1	Heat shock protein family E (Hsp10) member 1
ICGC	International Cancer Genome Consortium
IGFBP	Insulin-like growth factor binding protein
KIF	Kinesin family protein
LATS2	Large tumor suppressor kinase 2
lncRNA	Long non-coding RNA
LP-WGS	Low-pass whole genome sequencing
LRP4	LDL Receptor Related Protein 4
MAD2L1	Mitotic arrest deficient 2 like 1
MDM4	MDM4 regulator of p53
Merlin	Moesin-ezrin-radixin-like protein
MET	Mesenchymal-to-epithelial
miRNA	MicroRNA
MM	Mithramycin
MMP	Matrix metalloprotease
MPM	Malignant pleural mesothelioma
MSAC	Mitotic spindle assembly checkpoint
MSLN	Mesothelin
mTORC1/2	mTOR complex 1/2
MYC	MYC proto-oncogene, bHLH transcription factor
NC	NF2 and CDKN2AB
NCI	National Cancer Institute
ncRNA	Non-coding RNA
NDC80	Kinetochore complex component
NF2	Neurofibromatosis type 2
NGS	Next generation sequencing
NHGRI	National Human Genome Research Institute
PDGF	Platelet-derived growth factor
PDX	Patient-derived xenograft
PFS	Progression-free survival
PI3K	Phosphoinositide 3-kinase
PKC	Protein kinase C
PLK1	Polo like kinase 1
pRb	Retinoblastoma protein
PRC2	Polycomb repressive complex 2
PTCH1	Patched 1
RAN	RAN, member RAS oncogene family
RNAi	RNA interference
RNR	Ribonucleotide reductase
RPS15A	Ribosomal protein S15a
RPTOR	Regulatory associated protein of mTOR complex 1
RRM1	Ribonucleotide reductase catalytic subunit M1
RRM2	Ribonucleotide reductase catalytic subunit M2
RSTK	Receptor serine/threonine kinase
RTK	Receptor tyrosine kinase
RUVBL1	RuvB like AAA ATPase 1
SETD2	SET domain containing 2
SFK	Src Family Kinase
SMRP	Soluble mesothelin-related peptides
SNRNP	Small nuclear ribonucleoprotein
SNRPD1	Small nuclear ribonucleoprotein D1 polypeptide
SP1	Sp1 transcription factor
SUZ12	SUZ12 polycomb repressive complex 2 subunit
TCGA	The Cancer Genome Atlas
TERT	Telomerase reverse rranscriptase
TF	Transcription factor
TGF-β	Transforming growth factor β
TP53	Tumor Protein P53
TS	Thymidylate synthase
TTN	Titin
UBA1	Ubiquitin like modifier activating enzyme 1
UCH	Ubiquitin carboxy-terminal hydrolase
VCP	Valosin containing protein
VEGF	Vascular endothelial growth factor
WT1	Wilms Tumor Protein 1
YAP1	Yes-associated protein
ZEB1	Zinc finger e-box binding homeobox 1
